# A composite biomarker using multiparametric magnetic resonance imaging and blood analytes accurately identifies patients with non-alcoholic steatohepatitis and significant fibrosis

**DOI:** 10.1038/s41598-020-71995-8

**Published:** 2020-09-17

**Authors:** Andrea Dennis, Sofia Mouchti, Matt Kelly, Jonathan A. Fallowfield, Gideon Hirschfield, Michael Pavlides, Rajarshi Banerjee

**Affiliations:** 1 Perspectum, Gemini One, 5520 John Smith Drive, Oxford, OX4 2LL UK; 2 Centre for Inflammation Research, University of Edinburgh, Edinburgh, UK; 3 Toronto Centre for Liver Disease, University Health Network, Toronto, Canada; 4 Radcliffe Department of Medicine, University of Oxford, Oxford, UK

**Keywords:** Diagnostic markers, Hepatology, Drug development, Biomarkers

## Abstract

Non-alcoholic steatohepatitis (NASH) is major health burden lacking effective pharmacological therapies. Clinical trials enrol patients with histologically-defined NAFLD (non-alcoholic fatty liver disease) activity score (NAS) ≥ 4 and Kleiner-Brunt fibrosis stage (F) ≥ 2; however, screen failure rates are often high following biopsy. This study evaluated a non-invasive MRI biomarker, iron-corrected T1 mapping (cT1), as a diagnostic pre-screening biomarker for NASH. In a retrospective analysis of 86 biopsy confirmed NAFLD patients we explored the potential of blood and imaging biomarkers, both in isolation and in combination, to discriminate those who have NAS ≥ 4 and F ≥ 2 from those without. Stepwise logistic regression was performed to select the optimal combination of biomarkers, diagnostic accuracy was determined using area under the receiver operator curve and model validated confirmed with and fivefold cross-validation. Results showed that levels of cT1, AST, GGT and fasting glucose were all good predictors of NAS ≥ 4 and F ≥ 2, and the model identified the combination of cT1-AST-fasting glucose (cTAG) as far superior to any individual biomarker (AUC 0.90 [0.84–0.97]). This highlights the potential utility of the composite cTAG score for screening patients prior to biopsy to identify those suitable for NASH clinical trial enrolment.

## Introduction

Non-alcoholic fatty liver disease (NAFLD), is one of the most common forms of chronic liver disease, and a component of the metabolic syndrome, affecting 60% to 70% of patients with type 2 diabetes^1–3^. Non-alcoholic steatohepatitis (NASH) is a more progressive subtype of NAFLD with a 3–12% prevalence^4,5^, and is predicted to become the leading aetiology for liver transplantation^6^. NASH is characterised histologically by the presence of hepatocyte ballooning degeneration, hepatic lobular inflammation and presence of hepatic steatosis with patients at increased risk of fibrosis and progression to cirrhosis, hepatocellular carcinoma, cardiovascular disease, and death^7^. The first pharmacological treatment for NASH, the FXR agonist obeticholic acid, is expected to receive regulatory approval in 2020^8^, but there are a further 5 drugs in phase III clinical trials with approximately 130 active trials in total. The NAFLD Activity Score (NAS), proposed by the NASH Clinical Research Network (NASH-CRN) is one of the most frequently used histological scoring systems in NASH clinical trials. NAS is derived by summing the histological staging for liver fat (stage 0–3), lobular inflammation (stage 0–3), and ballooning (stage 0–2), with a NAS of 4 or greater typically regarded NASH^9^. While fibrosis stage does not form part of the NAS, enrolment criteria for NASH clinical trials now typically requires patients to not only have a NAS ≥ 4 but also evidence of fibrosis of stage 2 or more (scored using the Kleiner-Brunt (0–4) scale. This is as a result of rapidly evolving understanding of disease pathology, progression, and a growing body of evidence to support the bi-directional nature of fibrosis and its prognostic value in both liver-related and overall mortality, independent of NASH^10,11^. Identifying patients most likely to meet the NASH-CRN enrolment criteria on biopsy is an on-going challenge, with existing clinical indicators lacking both sensitivity and specificity. As a result, NASH trials often suffer from high levels of screen failure following central review of the baseline liver biopsy. In fact, 73% and 65% of biopsied subjects from PIVENS and CENTAR NASH clinical trials, respectively, did not meet the eligibility criteria^12,13^. Pre-biopsy enrichment strategies are becoming increasingly popular to reduce the number of screen failures and metrics derived from magnetic resonance imaging (MRI) such as iron-corrected T1 (cT1)-mapping and proton density fat fraction (PDFF) are emerging as promising non-invasive diagnostic biomarkers in NASH.

T1 mapping has shown promise as an effective imaging biomarker of inflammation (I) and fibrosis in myocardium^14,15^ and liver^16^, and as a prognostic biomarker for predicting clinical outcomes in chronic liver disease patients^17,18^. The presence of iron however, which can be accurately measured from MRI-T2star relaxation time (T2*), opposes the MR signal and *artificially* shortens the T1 relaxation time^19^, thus needs to be accounted for. Liver*MultiScan* (Perspectum Ltd, UK) quantifies iron-corrected T1 (cT1)^20^, by removing the confounding effect of elevated iron on liver T1. Liver cT1 has been shown to correlate with the hallmarks of fibro-inflammatory disease^16,20,21^ and shows promise as a risk stratification tool in NASH^16^. PDFF distinguishes the proportion of the MR-visible protons due to fat from all MR-visible protons (attributable to both fat and water) expressed as a percent, and has been widely reported to correlate very well with histologically graded liver steatosis^22–28^. Despite PDFF not routinely being reported to show correlation with the other histological features of NASH, and in fact decreases with advancing fibrosis^27,29^, it has been well accepted by the NASH community as a biomarker for clinical trials, both as a screening tool to identify suitable participants for trial enrolment, and as an exploratory endpoint for treatment efficacy^30,31^.

Many biomarkers have been investigated to identify NASH patients including clinical, biochemical, metabolic, and lipid analytes that predict some of the molecular mechanisms of the pathogenesis and progression of NAFLD. NAFLD pathogenesis is complex and it is very unlikely that a single biomarker can reliably distinguish NASH^32^. With this in mind, this study aimed to investigate the diagnostic accuracy of image-derived biomarkers and a host of circulating biomarkers that were acquired as part of standard care, both independently and in combination, for identifying NAFLD patients with clinical trial enrolment criteria of NAS ≥ 4 and F ≥ 2.

## Methods

### Study design, setting

This was a retrospective analysis of data combined from two prospective, cross-sectional studies into the utility of MRI methods to evaluate liver disease. The CALM study^33^ invited adult patients scheduled for a standard-of-care liver biopsy to investigate known or suspected liver disease from two large tertiary UK liver centres (Queen Elizabeth Hospital Birmingham and Royal Infirmary of Edinburgh) between February 2014 and September 2015. The RIAL/NICOLA study^34^ invited all patients referred for liver biopsy at two UK study centres (Oxford and Reading) between March 2011 and May 2015 to take part. Patient exclusion criteria were inability or unwillingness to give fully informed consent, any contraindication to MRI, and liver biopsy targeted at a focal liver lesion. For the purpose of this analysis only those patients with a primary diagnosis of either NAFLD or NASH who had not undergone liver transplantation were included. Patients underwent standard of care liver function blood tests and liver biopsy, and also underwent Liver*MultiScan* to measure cT1 and PDFF. We refer to the combination of RIAL/NICOLA and CALM data sets as the ‘original dataset’.

Both the studies were conducted in accordance with the ethical principles of the Declaration of Helsinki 2013 and Good Clinical Practice Guidelines. The RIAL study was approved by the institutional review departments at the University of Oxford and by the National Review Ethics Service (South Central; Ref: 11/H0504/2). The CALM study was approved by the institutional review departments at the University of Birmingham and by the National Review Ethics Service (West Midlands—The Black Country; Ref: 14/WM/0010). All participants gave written informed consent. The RIAL study was registered with clinicaltrials.gov (NCT01543646) and was sponsored by the University of Oxford. The CALM study was registered with the International Standard Randomised Controlled Trial Number registry (ISRCTN39463479) and the National Institute of Health Research portfolio (15,912). The study sponsor was the University of Birmingham.

### Histological analysis of liver biopsy samples

All biopsies were reported by at least 2 liver histopathologists for both studies^34,35^, and adequacy assessed using the definition of the Royal College of Pathologists^36^. Histology was graded according to the NASH-CRN for Kleiner-Brunt Fibrosis; hepatocellular ballooning; lobular inflammation; steatosis and the composite NAS. All pathologists were blinded to patient characteristics and non-invasive assessment data. Biopsy scores used for the analysis were those collected as part of the three independent studies and were not re-read centrally.

### Magnetic resonance imaging protocol

**Figure 1:**
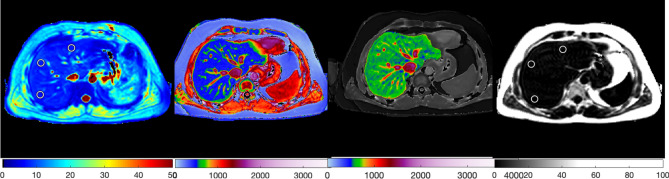
Example T2*, uncorrected T1, corrected T1 (cT1) and PDFF maps (from left to right) acquired using the Liver*MultiScan* protocol and generated using Liver*MultiScan* Version 3.1 software (Perspectum, Oxford, https://perspectum.com/products/livermultiscan).

The Liver*MultiScan* MRI scanning protocol was installed, calibrated and phantom tested on all the MR systems in these trials in a standard way^37^. Patients underwent their MRI (SIEMENS MAGNETOM TrioTim, Magnetic Field Strength 3 T) having fasted for at least 4 h. The average scan time for this protocol was 10 min. The protocol included a shortened modified look-locker inversion recovery (ShMOLLI, [TR 234.97 ms; TE 1.02 ms; FA 35; Slice Thickness 8 mm]) sequence to derive T1-relaxation and multi-echo spoiled-gradient-echo chemical shift encoded acquisition (DiXON_T2Star_GRE [TR 600 ms; TE 2.46 ms, 7.38 ms, 12.3 ms, 17.22 ms, 22.14 ms; FA 20; Slice Thickness 3 mm]) to calculate T2* and PDFF maps in most cases, although some PDFF values were generated using in vivo proton magnetic resonance spectroscopy (MRS), a specialised magnetic resonance technique that measures fat by quantifying the overall volume fraction of lipids in the liver parenchyma. Four single transverse slices were captured through the liver centred on the porta hepatis. Anonymised MR data were analysed off-site using Liver*MultiScan* software by image analysts trained in abdominal anatomy and artefact detection, who were blinded to the clinical data and risk grouping. For T2* (measured in milliseconds, ms) and PDFF (measured in %), three 15 mm diameter circular regions of interest (ROIs) were selected on the transverse maps to cover a representative sample of the liver parenchyma. For cT1 (ms) ROIs were placed on the central slice within the typical percutaneous biopsy region. Median values from all pixels within the ROIs were calculated and used as the representative score. Example cT1 and PDFF maps acquired using the Liver*MultiScan* protocol are displayed in Fig. 1.

### Statistical analysis

Statistical analysis was performed using R software version 3.5.3^38^ and a p-value less than 0.05 was considered statistically significant. Case-wise deletion was employed to include only complete cases for NAS, Kleiner-Brunt Fibrosis score and cT1 and PDFF, ALT, AST, albumin, bilirubin, GGT, and fasting blood glucose scores. Descriptive statistics were used to summarise baseline participant characteristics. Mean and standard deviation (SD) were used to describe normally distributed continuous variables, median with interquartile range for non-normally distributed, and frequency and percentage for categorical variables. Mean difference in biomarker values between those with NAS ≥ 4 and F ≥ 2 on biopsy versus those without, were compared by Student t-test with common variance and Fisher’s exact test, respectively.

To discriminate patients with progressive NASH, defined as having a NAS ≥ 4 (in the presence of ballooning ≥ 1 and lobular inflammation ≥ 1), with Kleiner-Brunt Fibrosis ≥ 2, from NAFLD patients not meeting these criteria, univariable logistic regression analysis was performed for all the potential predictors which included cT1, PDFF; and the serum measures acquired as part of standard clinical care: fasting glucose, AST, GGT, ALT, albumin and bilirubin. Following this, stepwise logistic regression analysis was performed using Akaike information criterion^39^ to select the optimal combination of MRI and blood serum derived predictors (model 1). All potential biomarkers were normalised using the z-score (linearly transformed data values having a mean of zero and standard deviation of 1), to allow for a meaningful interpretation because of the differences in the range and magnitude of the different units for all biomarkers. Risk scores were extracted from the odds ratio estimates of having NAS ≥ 4 and F ≥ 2 as calculated in the logistic regression model. Overall diagnostic accuracy produced by individual metrics and model 1 was estimated as the area under the receiver operator curve (AUC) with 95% CI.

Model 1 was validated using fivefold cross-validation by randomly splitting into 5 equal subsamples; 4 subsamples were used to train the model and the one left to test the model. This process was repeated 5 times, so every subsample was used once as a test dataset. The AUC was extracted as the mean across the 5 estimates from the fivefold cross-validation method. To investigate the potential effect of age and gender on discriminating patients with NAS ≥ 4 and F ≥ 2 from those without, model 1 was further adjusted (model 2) as a sensitivity analysis. The Wald test was used^40^ to investigate if significant improvement was added to the fit of model 1, when age and gender were included; and DeLong’s non-parametric test^41^ was used to compare the overall diagnostic performance between nested models 1 and 2.

## Results

**Figure 2:**
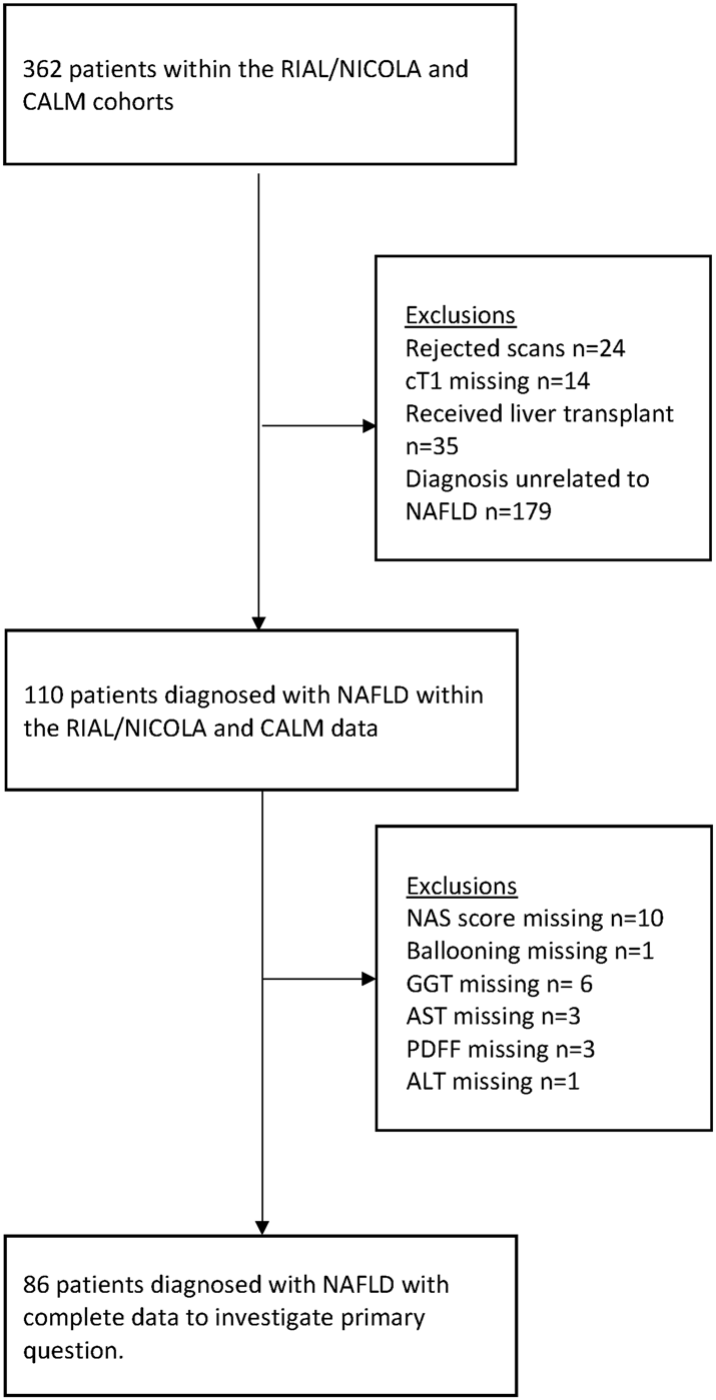
Flow diagram of patient inclusion in the RIAL/NICOLA and CALM data.

362 biopsied patients were initially included in the dataset. After applying the exclusion criteria, 86 patients were included in the analysis (Fig. 2). Mean interval between biopsy and MRI was 66 days (SD: 86 days, range 0–311). 39.5% of patients were classified in the NAS ≥ 4 and F ≥ 2 group (Table 1). The blood serum metrics of bilirubin, albumin, GGT, and ALT had similar distributions in both groups. AST and fasting glucose were significantly higher in the NAS ≥ 4 and F ≥ 2 group (p = 0.001 and p < 0.0001, respectively). The mean cT1 was significantly higher in patients classified as NAS ≥ 4 and F ≥ 2 compared to those without these criteria (934 ms vs. 854 ms; p-value = 0.0006, Table 1). PDFF did not differ significantly between the two groups.

**Table 1:** Descriptive statistics of demographic, serum and MRI metrics, described as mean and standard deviation for continuous variables or numbers and percentages for ordinal. Significant differences between those with NAS≥4 and F≥2 and those those with NAS<4 or F<2 are considered when p-value < .05, and are highlighted in bold.

	NAS < 4 or F < 2	NAS ≥ 4 and F ≥ 2	p-value
N (%)	52 (60.0)	34 (39.5)	
Age (years; mean [SD])	47.4 [13.3]	55.2[10.4]	**0.0029**
Sex (F, %)	23 (44.2)	14 (41.2)	0.8265
BMI (kg m^−2^; median; IQR)	34.6 ( 8.3)	33.8 (5.2)	0.5748
**Fibrosis (n, %)**			
F0	8 (15.4)	0 (0.0)	**< 0.0001**
F1	25 (48.1)	0 (0.0)	
F2	8 (15.4)	12 (35.3)	
F3	8 (15.4)	15 (44.1)	
F4	3 (5.8)	7 (20.6)	
**Ballooning (n, %)**			
B0	24 (46.2)	0 (0.0)	**< 0.0001**
B1	23 (44.2)	12 (35.3)	
B2	5 (9.6)	22 (64.7)	
**Lobular inflammation (n, %)**			
I0	23 (41.1)	0 (0.0)	** < 0.0001**
I1	32 (57.1)	7 (63.6)	
I2	1 (1.8)	3 (27.3)	
I3	0 (0.0)	1 ( 9.1)	
**Steatosis (n, %)**			
S0	3 (5.8)	0 (0.0)	**< 0.0001**
S1	21 (40.3)	8 (23.5)	
S2	8 (15.4)	15 (44.1)	
S3	20 (38.5)	11 (32.4)	
NAS* (n, %)			
NAS ≥ 4	22 (42.3)	34 (100)	**< 0.0001**
cT1 (mean ms; [SD])	854.4 (100.7)	934.2 (100.9)	**0.0006**
PDFF (mean %; [SD])	9.9 (7.8)	12.6 (7.4)	0.1111
Total bilirubin (µmol L^−1^)	14.3 (10.2)	14.7 (11.3)	0.8427
Albumin (g L^−1^)	43.9 ( 4.7)	45.2 ( 4.4)	0.1931
GGT (IU l^−1^)	83.9 (105.1)	134.1 (104.5)	**0.0330**
ALT (IU L^−1^)	59.0 (42.7)	69.5 (36.9)	0.2313
AST (IU L^−1^)	37.4 (15.1)	56.8 (29.6)	**0.001**
Fasting blood glucose (mmol L^−1^)	5.4 ( 1.3)	8.1 ( 3.4)	**< 0.0001**

**Figure 3:**
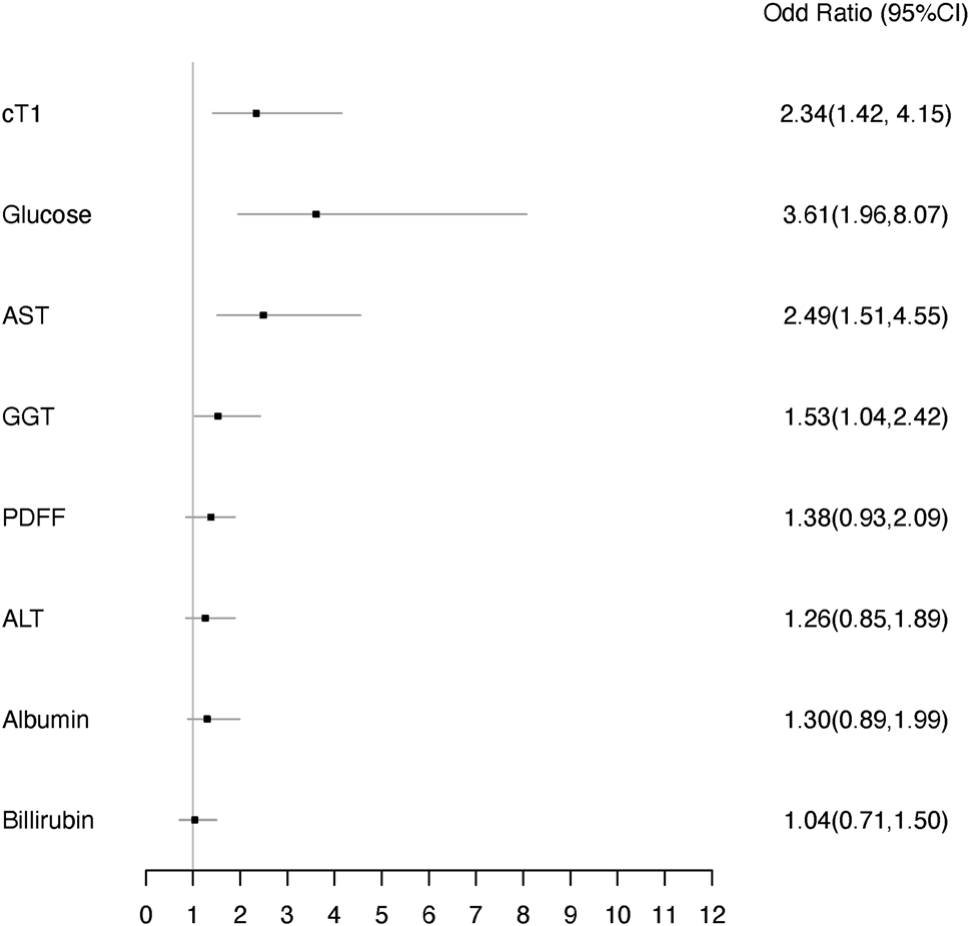
Forrest plot showing the odds ratios and 95% CI of the univariable logistic regression. All the variables were normalised (with z-scores).

Univariate analysis of the discriminatory ability of each of the biomarkers revealed a one unit increase in the normalised cT1, fasting glucose, AST and GGT, significantly increased the odds of having NAS ≥ 4 and F ≥ 2 by 2.34 (95% CI 1.42–4.15), 3.61 (95% CI 1.96–8.07), 2.49 (95% CI 1.51–4.55) and 1.53 (95% CI 1.04–2.42), respectively. Figure 3 illustrates the odds ratios of this univariable analysis. Stepwise multivariable logistic regression selected cT1, fasting glucose, and AST as the optimal combination to predict NAS ≥ 4 and F ≥ 2. Model 1 showed that a one unit increase in the normalised cT1, fasting glucose, and AST had a significant increase of 2.40 (95% CI 1.21–5.30), 4.56 (95% CI 2.34–11.67) and 3.13 (95% CI 1.62–7.20), respectively, in the odds ratio of having NAS ≥ 4 and F ≥ 2.

**Figure 4:**
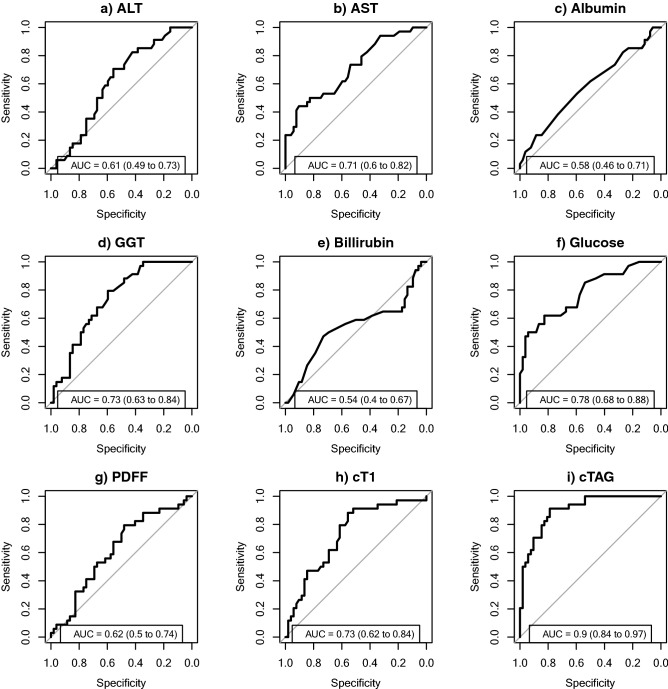
Receiver operating characteristic curves and AUROC with 95% CI for distinguishing NAS ≥ 4 and F ≥ 2. (**a**–**h**) illustrate the diagnostic performance of the individual biomarkers and (**i**) of the composite cTAG score (model 1) (n = 86 patients).

**Figure 5:**
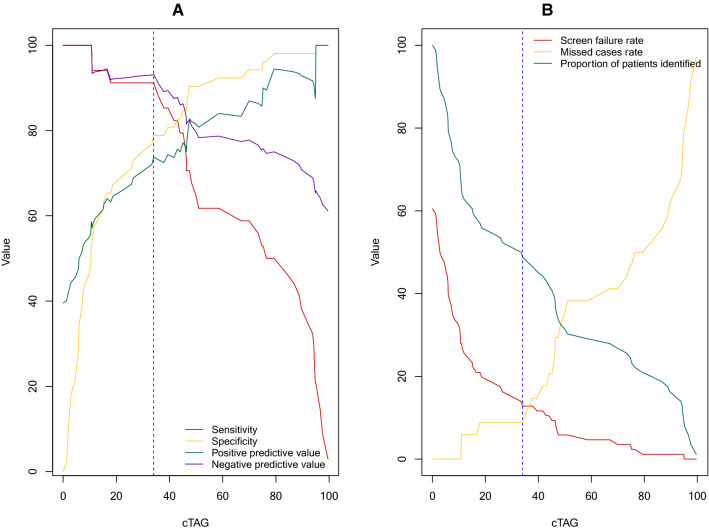
Diagnostic performance of cTAG for distinguishing NAS ≥ 4 and F ≥ 2. Vertical blue dashed line indicates the 34% cTAG cut-off presented in Table 2. (**A**) Sensitivity, specificity, positive predictive value, and negative predictive value for all possible cTAG score values (**B**) Screen failure rate, missed case rate, and proportion of patients identified for all possible cTAG score values.

The individual biomarkers cT1, AST, and fasting glucose yielded overall diagnostic performance of 0.73 (95% CI 0.62–0.84), 0.71 (95% CI 0.6–0.82) and 0.78 (95% CI 0.68–0.88), respectively (Fig. 4), in discriminating those with NAS ≥ 4 and F ≥ 2. The composite of the three biomarkers, abbreviated as the “cTAG” risk score (as extracted by model 1), yielded the highest diagnostic performance of 0.90 (95% CI 0.84–0.97) (Fig. 4). The diagnostic accuracy and test performance characteristics (sensitivity and specificity) for all potential cTAG cut-offs are displayed in Fig. 5. Selecting a cut-off from the model to achieve at least 90% sensitivity, yielded 34%; this gave a sensitivity of 92% and a specificity of 79% (Table 2). Cut-offs for the individual biomarkers in cTAG, that represent 90% sensitivity and 90% specificity are available in Tables S1 and S2 in the supplementary material respectively.

**Table 2:** Confusion matrix to discriminate NAS ≥ 4 and F ≥ 2 patients, using a cTAG risk score of 34% as a cut-off.

N = 86	NAS < 4 or F < 2	NAS ≥ 4 and F ≥ 2	
Risk score < 34%	41	3	NPV = 94%
Risk score ≥ 34%	11	31	PPV = 74%
	Sp. = 79%	Se. = 92%	

The negative predictive value (NPV) and positive predictive values (PPV) are illustrated too.

Model 1 resulted in the following equation for the cTAG risk score of having NAS ≥ 4 and F ≥ 2. cT1, AST and fasting glucose (Gluc) are on the normalised scale.

$${\text{Risk Score}}_{{{\text{NAS}} \ge 4\& {\text{F}} \ge 2}} = \frac{{{\exp}\left( { - 1.5 + 0.88{\text{cT}}1 + 1.14{\text{AST}} + 1.52{\text{Gluc}}.} \right)}}{{1 + {\exp}\left( { - 1.5 + 0.88{\text{cT}}1 + 1.14{\text{AST}} + 1.52{\text{Gluc}}.} \right)}},$$

### Sensitivity analysis: potential effect of age and gender

Model 2 showed that a one unit increase in the normalised age, and being male increased the odds of having NAS ≥ 4 and F ≥ 2 by 1.17 (95% CI 0.96–3.26) and 1.11 (95% CI 0.29–4.24), respectively; however, neither of these were statistically significant, while one unit increase in cT1, fasting glucose and AST, significantly increased the odds of having NAS ≥ 4 and F ≥ 2 by 2.54 (95% CI 1.27–5.26), 3.85 (95% CI 1.98–9.69) and 3.36 (95% CI 1.67–3.85) respectively. There was insufficient evidence to suggest that adding age and gender to the cTAG score discriminates more accurately those with NAS ≥ 4 and F ≥ 2 (Wald test between nested model 1 and 2, (p = 0.7690); DeLong test between the AUROC of the risk scores produced by models 1 and 2 (p = 0.76).

### Model validation

**Figure 6:**
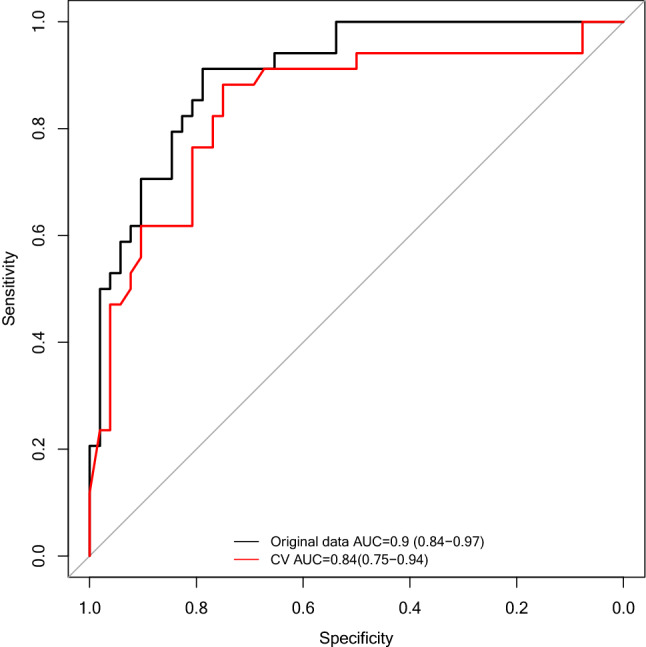
ROC curves drawn from the risk scores extracted for model 1 to discriminate NAS ≥ 4 and F ≥ 2 from those without from (i) the original cohort (black lines) and (ii) fivefold cross-validation (red lines).

The overall discriminative performance of model 1 in the fivefold cross-validation was AUROC = 0.84 (95% CI 0.75–0.94) (Fig. 6).

### cTAG for trial enrichment

The potential impact of screen failure rate, defined as the proportion of individuals who pass through screening but do not meet the histological criteria of NAS ≥ 4 and F ≥ 2 after biopsy, was explored for all potential cut-offs (Fig. 5). In a worked example, a cTAG risk score of 34% resulted in a screen failure rate of 13%, compared to 61% without. Using the same cTAG cut-off, 26% of patients that received a positive result failed to meet the histological criteria (false discovery rate, is defined as 100%-PPV). The selection of the optimal cut-off for trial enrichment will ultimately depend on the required balance between screen fail and missed cases rate.

## Discussion

In this retrospective cohort study, we highlight the utility of a novel composite score, cTAG, that combines cT1, a non-invasive MRI-derived biomarkers of liver disease with standard serum biomarkers, to identify patients with progressive NASH. In line with previously-used definitions of this high-risk NASH group^42^, and common enrolment criteria for NASH clinical trials (e.g. Regenerate NCT02548351; Resolve-IT NCT02704403; Maestro-Nash NCT03900429) we have targeted the identification of patients with histologically-determined NAS ≥ 4 and F ≥ 2.

In this study, fasting glucose, AST, GGT and the MRI biomarker cT1 each had good diagnostic performance to discriminate those with progressive NASH. Somewhat surprisingly given its common use as a screening tool and endpoint in NASH trials, PDFF demonstrated poor performance in discriminating these patient groups. This may be explained by the observed reduction in PDFF with advanced fibrosis which drops significantly after F3 thus diverging from the positive relationship^27,29^. Of the imaging and serum markers evaluated, the optimal combination: cT1, AST and fasting glucose (cTAG), demonstrated excellent performance in identifying patients with NAS ≥ 4 and F ≥ 2 (AUROC = 0.90) in this dataset, which was corroborated with cross-validation (AUROC = 0.84). Modelling the impact of using the cTAG score to enrich the population selected for liver biopsy revealed a reduction in screen failure rate from 61 to 13%, corresponding to an 87% higher chance of a selected patient meeting the histological criteria of NAS ≥ 4 and F ≥ 2. This highlights a potential role for this composite biomarker in both NASH clinical trials to reduce the number of avoidable invasive liver biopsies, and in secondary clinical care to evaluate NASH status. Health economic modelling has demonstrated Liver*MultiScan* to be cost effective for the detection of patients with NASH^35^, a value that is likely to even greater if the full cost of liver biopsy, accounting for complications was considered. Further research into the cost implications of non-invasive biomarkers for NASH in a variety of healthcare settings is warranted.

The utility of cT1 in distinguishing between these groups derives from the significantly higher cT1 in the progressive NASH group. To put the 79.8 ms difference in context, the reported standard deviation for cT1 reproducibility (same patient scanned across different MRI scanners) is 41.4 ms^37^ and 31.9 ms for a longitudinal test–retest study over 16 weeks^43^. Although the data reported in this study is not longitudinal, the magnitude of the difference between the two risk groups, relative to the reported repeatability and reproducibility, supports the utility of cT1 as a sensitive biomarker for monitoring changes in disease state^44^. Regarding the observed results for the blood-based biomarkers, the usual pattern of abnormal liver enzymes due to NAFLD is increased transaminases, with alanine aminotransferase (ALT) levels exceeding those of aspartate aminotransferase (AST). As NAFLD progresses to NASH and fibrosis the AST may increase and lead to a resultant rise in the ratio of AST/ALT^45,46^; the γ glutamyl-transferase (GGT) level can also increase. Although ALT and AST are useful tests, they are not reliable in predicting NAFLD. It has been found that up to 50% of NAFLD patients can have normal levels of AST and ALT^47,48^. Similarly, insulin resistance is a factor associated with NASH^49^, HOMA-IA for example has been identified as an independent predictor of advanced fibrosis in patients with NAFLD^50^ and the prevalence of NAFLD is estimated to be 60% in patients with type 2 diabetes^1,2^. The liver is also the main location of glucose production during fasting conditions thus fasting glucose a good marker of insulin resistance. Patients with hepatic steatosis may have increased fasting glucose^51^ but not always. These fluctuating patterns of the biomarkers in NASH highlight the added potential of augmenting the information available by combining imaging and circulating biomarkers to build a more accurate profile of underlying liver disease, an area that is rapidly evolving as evidenced by the wealth of emerging research into combined biomarkers (e.g. FAST^42^, ADAPT^52^). The promise of such approaches may not only match the prognostic performance of liver biopsy but likely one day surpass it by forming a basis for precision medicine.

Whilst the results of this analysis are very promising, the research is not without its limitations. The role of a single fasting glucose measurement to act as a predictor of NAS ≥ 4 and F ≥ 2 can be an inferior predictor compared to some other indicator of glycaemic control such as HbA1c, HOMA-IA, previous diagnosis of diabetes or current usage of antidiabetic medications, but none of these were consistently collected in the analysed dataset. Fasting glucose varies after meals, exercising or antidiabetic medications and this whilst it is common for glucose measurements to be assumed fasting, they may in fact reflect recent food intake, which can introduce bias. However, other studies suggested that fasting glucose is higher in patients with NASH, highlighting the potential use as a predictor of coexisting diabetes and NASH^51,53^. Another consideration that should be noted in studies of this nature is the potential for discordance with biopsy results as a result of a large time interval between measurements. The mean time interval for this study was 66 days, in which one would not expect any major change in the health of the liver in the absence of pharmaceutical intervention; however, in some cases the interval was up to 10 months. Whilst fibrosis has been reported to take up to 7 years to progress^54^, these intervals may influence the interpretability of the data in this cohort. Further validation studies should aim to minimise this interval where possible. It should also be noted that the sample size analysed in this study was relatively small, and future studies with larger datasets are required to independently validate the diagnostic performance of the model.

## Conclusion

While the individual biomarkers of cT1, fasting glucose and AST yielded good discriminatory performance in identifying progressive NASH, a composite of all three, the cTAG score, greatly improved the performance. The non-invasive, objective and organ-specific nature of cT1 compliments these routinely measured blood analytes. Together, this highlights the potential utility of cTAG in identifying patients at increased risk of disease progression who would be suitable for pharmacological therapy, either as part of a clinical trial or in routine clinical practice as treatments become available. Further research, possible exploring more stable and accurate markers of insulin resistance, are warranted in order to validate the model in independent cohorts with larger sample sizes and varying disease prevalence.

## Supplementary information

Supplementary file 1.
