# Supplementary material for: A composite biomarker using multiparametric magnetic resonance imaging and blood analytes accurately identifies patients with non-alcoholic steatohepatitis and significant fibrosis
Source: Sci Rep. 2020 Sep 17;10:15308. doi: 10.1038/s41598-020-71995-8 (PMC7499258; doi:10.1038/s41598-020-71995-8)
Supplement: Supplementary file 1 [file 41598_2020_71995_MOESM1_ESM.docx]

# **A composite biomarker using multiparametric magnetic resonance imaging and blood analytes accurately identifies patients with non-alcoholic steatohepatitis and significant fibrosis**

Andrea Dennis^1*^, Sofia Mouchti^1^, Matt Kelly^1^, Jonathan A. Fallowfield^2^, Gideon Hirschfield^3^, Michael Pavlides^,4^, Rajarshi Banerjee^1^.

^1^Perspectum, Oxford, UK; ^2^Centre for Inflammation Research, University of Edinburgh, UK; ^3^ Toronto Centre for Liver Disease, University Health Network, Toronto, Canada; ^4^Radcliffe Department of Medicine, University of Oxford, UK;

*Corresponding author: Andrea Dennis, [andrea.dennis@perspectum.com](mailto:andrea.dennis@perspectum.com), +44(0)1865 655343, Gemini One, 5520 John Smith Drive, Oxford, OX4 2LL

Supplementary information

**Table 1. Biomarker cut-off that corresponded to 90% sensitivity with respective specificity, NPV and PPV.**

| **Biomarker** | **Cut-off** | **Spec. (%)** | **NPV (%)** | **PPV (%)** |
| --- | --- | --- | --- | --- |
| cT1 (ms) | 825 | 40 | 88 | 50 |
| Glucose (mmol.L-1) | 4.8 | 40 | 87 | 50 |
| AST (IU.L-1) | 29.5 | 37 | 83 | 48 |
| cTAG (%) | 34 | 79 | 93 | 74 |

**Table 2. Biomarker cut-off that corresponded to 90% specificity with respective sensitivity, NPV and PPV.**

| **Biomarker** | **Cut-off** | **Sens. (%)** | **NPV (%)** | **PPV (%)** |
| --- | --- | --- | --- | --- |
| cT1 (ms) | 980 | 26 | 64 | 65 |
| Glucose (mmol.L-1) | 6.9 | 50 | 73 | 77 |
| AST (IU.L-1) | 60.5 | 44 | 71 | 75 |
| cTAG (%) | 47 | 71 | 82 | 80 |
